# P-820. Impact on outcomes and detection of Infective Endocarditis in patients with *Staphylococcus aureus* bacteremia following an Infectious Disease Consultation: A Quasi-experimental study

**DOI:** 10.1093/ofid/ofae631.1012

**Published:** 2025-01-29

**Authors:** Deepak Kumar, Navneet Kaur, Gopal Krishna Bohra, Naresh Kumar Midha, Durga Shankar Meena, Surender Deora, Rahul Choudhary, Vibhor Tak, T R Neetha, Tejasvi kanagiri, Yash Khatod

**Affiliations:** All India Institute of Medical Sciences, Jodhpur (India), Jodhpur, Rajasthan, India; All India Institute of Medical Sciences, Jodhpur, Jodhpur, Rajasthan, India; AIIMS, Jodhpur, Rajasthan, India; AIIMS, Jodhpur, Rajasthan, India; AIIMS, Jodhpur, Rajasthan, India; All India Institute of Medical Sciences, Jodhpur, Jodhpur, Rajasthan, India; All India Institute of Medical Sciences, Jodhpur, Jodhpur, Rajasthan, India; AIIMS Jodhpur , Jodhpur, Rajasthan, India; AIIMS Jodhpur, Jodhpur, Rajasthan, India; AIIMS, Jodhpur, Jodhpur, Rajasthan, India; AIIMS Jodhpur, Jodhpur, Rajasthan, India

## Abstract

**Background:**

*Staphylococcus aureus* Bacteremia (SAB) is associated with high morbidity and mortality ranging from 10-40% with an in-hospital mortality of 13-50%. The primary concern is its ability to cause metastatic complications in as many as 53% of cases which contributes to poor prognosis. The positive impact of formal Infectious Disease consultation (IDc) in SAB management is a well-known concept in Western Countries.

The published data from developing countries is limited, with particularly no studies available from India.

The present study was undertaken to know the impact of ID consultation on outcomes and detection of IE in SAB patients

Workflow of ID Consultation in SAB cases

**Figure:**
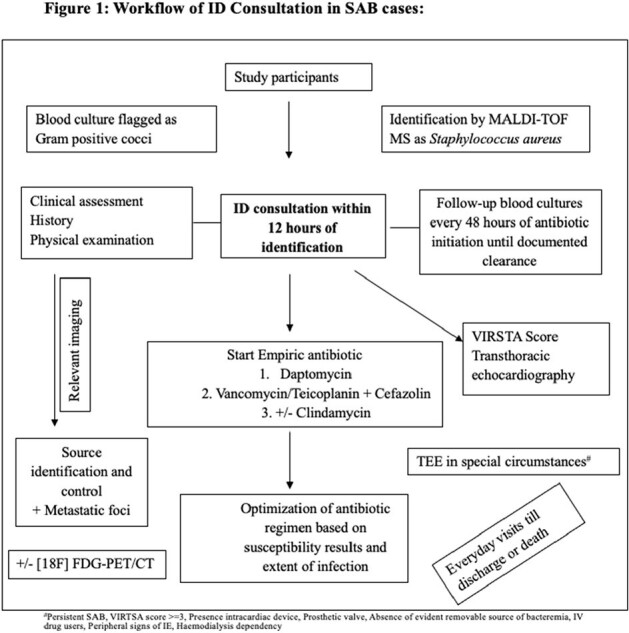

**Methods:**

This was a quasi-experimental study carried at a tertiary care centre of Western India after approval from Institutional Ethical Committee (IEC).

**Inclusion criteria**: All hospitalised patients with confirmed blood stream infections (BSI) due to *Staphylococcus aureus*

**Exclusion criteria**: Patients with Polymicrobial BSI, with documented SAB in previous 12 weeks and patients surviving ≤72 hours after positive index blood culture

The data of SAB with ID consultation was collected prospectively between January 2023-December 2023 and the data without IDc was collected retrospectively from January 2021 – December 2022. Informed consent was taken from every study participant; however, IEC waived the requirement for informed consent for the retrospective two-year data of SAB.

The primary and secondary outcomes were analyzed

The workflow of ID consultation is summarised in Fig. 1

Baseline characteristics of patients with Staphylococcus aureus bacteremia

**Figure:**
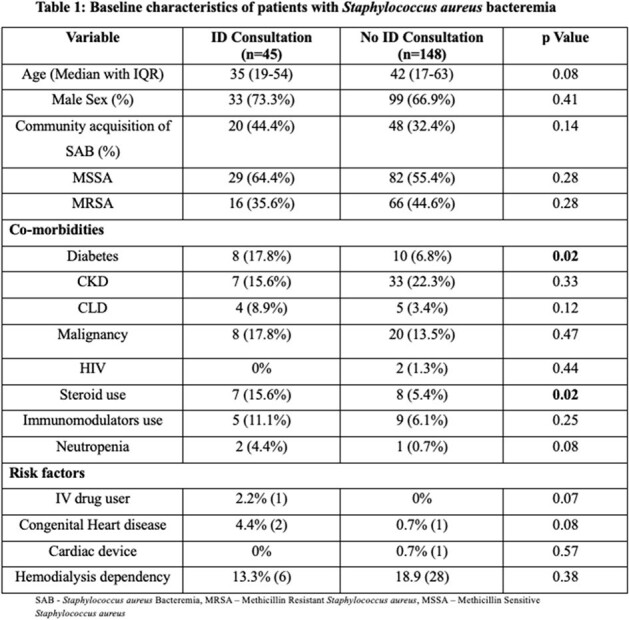

**Results:**

A total of 55 patients were diagnosed with SAB in IDc group and 45 were included in final analysis.

A total of 180 patients were diagnosed with SAB in retrospective data who had no access to IDc and 148 were included in final analysis.

Table 1 shows the comparison between demographic profile, co-morbidities and risk factors associated with SAB cases in IDc group vs no IDc group. Most of them were comparable except Diabetes mellitus and Steroid use which was higher in IDc group.

Table 2 depicts statistically significant difference between primary and secondary outcomes among IDc group in comparison to no IDc group.

Primary and Secondary outcomes in patients with SAB

**Figure:**
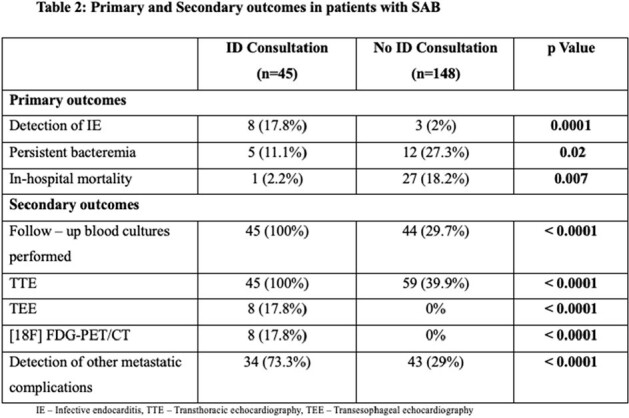

**Conclusion:**

Timely ID consultation in cases of SAB is prudent as this can help in streamlining the management and can bring out improved outcomes for patients.

**Disclosures:**

**All Authors**: No reported disclosures

